# Community and Voice: Emphasizing Black and Latine Adolescents' Strengths Promotes Identity Alignment, Belonging, and Academic Persistence

**DOI:** 10.1002/jad.70049

**Published:** 2025-09-09

**Authors:** Régine Debrosse, Ivan A. Hernandez

**Affiliations:** ^1^ McGill University Montreal Canada; ^2^ California Polytechnic State University San Luis Obispo California USA

## Abstract

**Introduction:**

The present research examined whether Black and Latine adolescents' academic persistence could be promoted through two novel strength‐based reflection activities, providing them an opportunity to experience a sense of school belonging and to form meaningful connections between their racial/ethnic identity and their ideal future identity they aspired for.

**Methods:**

A randomized‐controlled experiment was conducted in the U.S. with Black and Latine adolescents (*n* = 278, including 134 girls and 117 boys, *M* = 14.05 years old). Academic persistence was assessed by examining two markers: how much adolescents were engaged in school, as well as how much they interpreted school difficulties as indicating the importance of school.

**Results:**

Black and Latina girls assigned to the ‘community resourcefulness reflection’ who were invited to reflect on strategies and advice from their racial/ethnic communities (vs. their peers who were not) saw their racial/ethnic and ideal career identities as more aligned, which in turn was associated with increased academic engagement and increased likelihood of interpreting school difficulties as indicating school importance. Moreover, Black and Latine adolescents assigned to the “voice reflection” who were invited to reflect on how their voice could play a powerful role in spaces where they are underrepresented (vs. their peers who were not) reported more school belonging, which in turn was associated with increased academic engagement and increased likelihood of interpreting school difficulties as indicating school importance.

**Conclusions:**

These findings indicate that approaches focused on racial/ethnic strengths foster positive identity connections, school belonging, and academic persistence for adolescents of color.

## 1 Introduction

For young Black and Latine people in North America, adolescence is an especially important time to explore and establish what race and ethnicity mean for the way they understand themselves (Booth et al. 2022; Rivas‐Drake et al. 2014). Adolescence is a turning point for developing the multiple identities that represent different aspects of who adolescents are (Harter 2012; Kroger et al. 2010; Umaña‐Taylor et al. 2014). These different identities are sometimes referred to as different selves, different self‐aspects, or different components of one's identity; here, we will refer to each of these facets as an ‘identity.’ Identities include who adolescents say they are currently (i.e., their present identity), whom they aspire to become (i.e., their ideal future identity), and who they are in relation to their background and communities (e.g., their racial/ethnic identity). Additionally, adolescents also reflect on the *connections* between the different identities they hold and, relatedly, reflect on where they feel that they can *belong* as a function of who they are—both of which have implications for academic trajectories. Adolescents are more motivated and perform better academically when they form strong connections between their identities (Debrosse et al. 2018; Destin and Hernandez#x000A0;^2021^; Oyserman and Destin 2010), as well as when they feel that they belong in school (Gopalan and Brady 2020; Williams et al. 2020). Given that historical barriers impeded access to many opportunities, Black and Latine adolescents can be further hindered in their ability to connect their racial/ethnic identity with their ideal identity and to experience belonging in school.

The present research draws from identity‐based motivation and strength‐based theoretical frameworks to investigate two reflection activities designed to foster connections between adolescents' racial/ethnic identity and their ideal future identity, encourage school belonging, and improve academic persistence for Black and Latine adolescents. The first activity guides Black and Latine adolescents to reflect on advice and strategies from their racial/ethnic community that they can use when pursuing their aspirations. The second activity invites reflections on how adolescents can use their voices when their racial/ethnic group is underrepresented. The current randomized‐controlled experiment shows that highlighting strengths associated with their racial/ethnic experiences benefits Black and Latine adolescents academically, via pathways that forge identity connections or that develop their sense of school belonging.

### 1.1 Alignment of Racial/Ethnic and Ideal Identities and School Belonging

Many adolescents of color positively regard their racial/ethnic identity, and when they do, it can serve as a protective factor for them (Emuka and Karras 2024; Hope et al. 2019; Jones and Neblett 2016; Rivas‐Drake et al. 2014). Yet, Black and Latine adolescents can find it difficult to form strong connections between their racial/ethnic identity and their ideal future identity. This idea is captured by the term ethnic–ideal alignment for short, and it which includes connections between racial and ideal identities as well as connections between ethnic and ideal identities (Debrosse et al. 2024; Debrosse et al. 2020). Moreover, many students of color experience a lack of belonging in school settings, especially when they sense their identities and experiences are not all welcome, including their racial/ethnic experiences (Gray et al. 2018).

Low ethnic–ideal alignment and low school belonging can occur partly because Black and Latine adolescents contend with and can be undermined by negative stereotypes and deficit‐based narratives about their racial/ethnic identity, culture, and communities, which underplay their strengths (e.g., Valencia 2010; Yosso 2005). Black and Latine communities are generally depicted with limiting narratives and narrow stereotypes, and these depictions impede opportunities for thriving, especially in school settings (Silverman, Hernandez, et al. 2023; Starck et al. 2020; Steele et al. 2002). Thus, ethnic–ideal alignment is one way in which larger macro systems and their messages affect how adolescents view themselves (see Rogers et al. 2021). For instance, Black, Latine, and other adolescents of color tend to be more engaged in school and persevere in pursuing their future goals when they perceive strong ethnic–ideal alignment and conversely, less engaged in school when they perceive low ethnic–ideal alignment (Debrosse et al. 2018; Debrosse et al. 2020).

Besides sensing that their race/ethnicity aligns strongly or weakly with their aspirations, people's sense that they can fit in within contexts where they pursue their aspirations also matters (Schmader and Sedikides 2018), especially for their academic experiences (Allen, Gray, et al. 2022). While feeling that they belong at school is important to Black and Latine adolescents, it is often undermined by negative stereotypes and underrepresentation (Allen, Jamshidi, et al. 2022; Murphy and Zirkel 2015), as well as by fewer opportunities to connect (Gray et al. 2018; Voight et al. 2015). Yet, when Black and Latine students do experience equitable opportunities to belong, they benefit significantly through a range of outcomes such as improved academic persistence and increased well‐being (Gillen‐O'Neel et al. 2013; Gopalan and Brady 2020). In this context, successfully targeting ethnic–ideal alignment and school belonging could help counteract some of the effects of narrow and negative narratives.

### 1.2 Identity‐Based Motivation and Strength‐Based Approaches

Identity‐based motivation can help explain findings surrounding ethnic–ideal alignment and school belonging, even though neither notion emerged within the framework. When an important identity connects with a particular goal or aspiration, students tend not to interpret the difficulties in pursuing this goal or aspiration as cues to give up but, rather, as cues to double down on their efforts (see Destin and Hernandez 2021; Oyserman and Destin 2010). For example, adolescents invest more effort in school and obtain better academic outcomes when encouraged to connect their present with their ideal future identities and to draw plausible paths to their aspirations (Destin and Oyserman 2009; O'Donnell & Oyserman 2023). Thus, adolescents who see their racial/ethnic identity as disconnected from their future aspirations (i.e., with low ethnic–ideal alignment) or who are unsure that their full person belongs in school settings (i.e., with low school belonging) are less likely to engage and to interpret difficulties as indicating importance. For these reasons, identity‐based motivation suggests that increasing ethnic–ideal alignment and school belonging could influence adolescents' academic persistence, and eventually their academic paths—and it further suggests that supporting connections between identities and aspiration domains could help do so.

Strength‐based approaches provide specific pointers about how to foster ethnic–ideal alignment and school belonging for Black and Latine adolescents. Strength‐based approaches challenge the idea that people with marginalized backgrounds or experiences, their families, or their communities possess inherent deficits. Strength‐based approaches cannot be captured by a single unified framework or theory, or even from one area of psychology, though many refer to the work of Freire (1973) as seminal. Instead, strength‐based approaches encompass frameworks and theories that share the premise that marginalized people, families, and communities have strengths and assets that need to be highlighted and valued (Silverman, Rosario, et al. 2023). For example, some scholars posit that Black and Latine adolescents have acquired unique knowledge, strengths, and resources from their family and social networks, as well as from their lived experiences (Gonzalez et al. 2005; Moll et al. 1992; Yosso 2005). By focusing on people's identities as a source of motivation, the identity‐based motivation framework finds points of resonance with strength‐based approaches (Oyserman and Destin 2010).

Seminal work promoting strength‐based approaches has proposed that the assets, strengths, and experiences of marginalized people, particularly people of color, can be leveraged to bring desired changes in their lives (e.g., Freire 1973; Ladson‐Billings 1995). Inspired by their work and others', interventions and programs leveraging strengths have been successful with adolescents of color (e.g., Chun and Dickson 2011). For instance, reflecting on non‐stereotypical strengths from their community fostered Black and Latine adolescents' ethnic–ideal alignment (Debrosse 2023). Similarly, Hernandez et al. (2021) found that after reflecting on strengths they developed because of their lived experiences, Black and Latine adolescents persisted more academically and viewed themselves as assets to their schools and society. Emphasizing strengths can also foster school belonging (Soria and Stubblefield 2015).

Strength‐based approaches are effective for several reasons. First, they inherently push back on the essentialist and deficit‐based discourse so harmful to marginalized people, particularly in educational settings (Hernandez et al. 2021; Silverman, Rosario, et al. 2023). In focusing on strengths while acknowledging challenges, these approaches nuance and complexify narratives about marginalized people's experiences (Covarrubias and Laiduc 2022), which is especially important for the positive development of adolescents (Hoffman and Umaña‐Taylor 2023). Second, strength‐based approaches can explicitly highlight racial and ethnic experiences (Debrosse 2023); in fact, they often bring up oppression, systemic barriers, and challenges (Gonzalez et al. 2005; Yosso 2005). As such, they can provide paths to integrating racial/ethnic experiences in a way that can support adolescents of color without fostering internalized racism or otherwise harming them (see Neville et al. 2013). Third, strength‐based approaches build from people's dreams and hopes for themselves and from their understanding of their lives (A. Bauer et al. 2025). As they place marginalized folks at the heart of identifying strengths and leveraging them, the resulting idiosyncratic solutions can resonate deeply and thus be more conducive to change.

Therefore, strength‐based approaches are well‐positioned to counter the harmful narratives and stereotypes and to provide nuanced stories that resonate and successfully bring desired change (e.g., A. Bauer et al. 2025; Silverman, Rosario, et al. 2023). Race‐related, strength‐based reflections could improve academic persistence in Black and Latine adolescents by supporting connections between their racial/ethnic and ideal future identities and by fostering school belonging. Opportunities to reflect on unique strengths associated with their otherwise marginalized background should be particularly effective (Hernandez et al. 2021; Silverman, Rosario, et al. 2023). In this way, Black and Latine adolescents can draw from and leverage their own cultural capital as well as the knowledge and strategies of their community. Strength‐based activities thus have the potential to support Black and Latine adolescents in terms of identity alignment, school belonging, and their academic persistence.

### 1.3 Gendered Racial/Ethnic Experiences in Schools

Being Black or Latine plays out differently depending on adolescents' gender experiences (Rogers and Syed 2021; Velez and Spencer 2018). Indeed, Black and Latine adolescents' experiences are shaped by the interaction between their unique intersecting identities and the systems of power and inequality that influence the educational environments that they navigate. For instance, some narratives, stereotypes, and expectations are distinct across gender, within the same racial/ethnic group. Empirically, Black and Latina adolescent girls sometimes experience school differently from Black and Latino adolescent boys. For example, girls' academic outcomes are impacted by inequities, race‐related messages, and community factors that do not impact boys' academic outcomes and vice versa (Butler‐Barnes et al. 2019; Cooper et al. 2022; Vera et al. 2018). Moreover, independently or through its modulation of racial/ethnic experiences, gender can produce distinct experiences in terms of school belonging (Allen et al. 2018) and identity (Butler‐Barnes et al. 2018; Velez and Spencer 2018). Thus, while there is no consistent pattern emerging from the literature regarding to the way in which they experience identity, community, race/ethnicity, and school, and while Black and Latine girls' and boys' experiences are sometimes similar (e.g., Rivas‐Drake et al. 2014; Sánchez et al. 2005), accounting for gender and its possible moderating effects is relevant.

### 1.4 The Present Study

The current research tests the benefits of two novel strength‐based reflection activities on the academic persistence of Black and Latine adolescents via their influence on ethnic–ideal alignment and sense of school belonging. First, building on research on identity‐based motivation theory (Destin and Hernandez 2021; O'Donnell & Oyserman 2023; Oyserman 2015), the community resourcefulness reflection invites adolescents to think about their ideal future identity and possible obstacles that could arise when pursuing it, while providing a list of strategies and advice from their communities for inspiration. This activity directly challenges pervasive and limiting discourse about Black and Latine communities, and invites Black and Latine adolescents to connect their communities' assets with their aspired paths. In so doing, this reflection fosters connections between adolescents' racial/ethnic and ideal future identities. Therefore, we expect that reflecting on community resourcefulness materials will improve markers of academic persistence (H1a), insofar as it increases ethnic–ideal alignment (H2a) or school belonging (H3a).

Second, building on different forms of knowledge and strengths, the voice reflection focuses on ways through which adolescents use their unique perspective, when they are one of the few people with their background in a given context. Similar to the community resourcefulness reflection, the voice reflection emphasizes racial/ethnic identities in the context of ideal future identities and elicits thoughts about positive avenues available to face obstacles related to these aspirations. Therefore, we expect that the voice reflection will improve markers of academic persistence (H1b), insofar as it increases ethnic–ideal alignment (H2b) or school belonging (H3b). Besides testing these hypotheses, the current research will also examine whether these two strength‐based reflection activities affect Black and Latine adolescent girls and boys differently.

## 2 Methods

### 2.1 Recruitment and Participants

Data were collected through a partnership with Character Lab, a consortium of researchers and educators that facilitates research in U.S. schools (see Duckworth 2019). Character Lab approved the project and the protocol we proposed, brought it to the attention of school partners, and determined sample size through systematic matching protocols. As requested, Character Lab matched this project with three predominantly Latine schools located in a diverse metropolitan area in Florida. In each partner school, adolescents participated in computers, using Qualtrics survey links. Character Lab then deidentified the data before sharing it with us (available upon request). Character Lab recruited 287 Black and Latine adolescents in schools where Latine students formed the largest racial/ethnic background represented. Analyses were not conducted with students who dropped out after starting the study (3.0% attrition: one for the no‐reflection neutral condition, four for the voice reflection condition, three for the community resourcefulness reflection condition).

The final sample is composed of 278 adolescents (134 identified as girls, 117 identified as boys; gender information was unavailable for 27 adolescents; *M* = 14.05 years‐old, SD = 1.08). In terms of race/ethnicity, 189 of the adolescents were Latine, 82 were Black, and 7 identified both Black and Latine or as Afro‐Latine (see for full demographic information and related statistics). In terms of socioeconomic status, 53.2% of participants received free or reduced lunch in school, whereas 22.3% did not (for the others, this information was not available).

### 2.2 Procedures and Measures

Students were randomly assigned to a condition by a computer. All conditions started by inviting participants to write about their ideal career/occupational aspiration and to list five “characteristics that describe the people who have this occupation, position, or job.” Then, depending on the condition to which they had been randomly assigned, they were either invited to complete the community resourcefulness reflection condition (*n* = 97), the voice reflection condition (*n* = 89), or they moved directly to dependent measures if assigned to the no‐reflection neutral condition (*n* = 92).

Participants assigned to the community resourcefulness reflection identified three strategies to surmount potential obstacles to their ideal future. A list of community strategies successfully used by Black and Latine people was provided for inspiration. The list was built by prompting Black and Latine adults to describe strengths they had witnessed within their community and themselves (see [REDACTED]). Their words were condensed in the list introduced with: *“*Everyone faces challenges and obstacles as they study in school or try to get their dream job. What kinds of strategies can you plan to use to surmount challenges when you study in school or try to get your dream job? Below, write down three strategies that you can think of. If it is useful to you, you can pick from or get inspired by the following strategies, which were listed by people in the Black and Latino/Latina/Latinx community when they reflected about what has helped them overcome challenges in school, in their job, and in life.”

Adolescents assigned to the voice reflection wrote about playing a positive role if in an underrepresented position: “as you get older and move toward adulthood, you may find yourself in spaces where no one or almost no one shares your background (e.g., it is possible that only a few people at your job have your racial/ethnic background). Although this reality is very common, many people find these situations quite challenging. However, your presence and point of view are powerful when you are one of the only ones of a certain background or with a certain experience. In these moments, other people have a lot to learn from the unique perspective that your voice brings to the table. Take a moment to think about your strengths and point of view. Write one way in which you feel that your voice could be powerful and have a positive impact as you move toward the job you dream of.”

#### 2.2.1 Ethnic–Ideal Alignment

Adolescents were asked whether each of the five ideal career identity characteristics described their racial/ethnic community by indicating their agreement on a scale from 1 (*strongly disagree*) to 5 (*strongly agree*), as in previous research (Debrosse et al. 2018; Higgins et al. 1997; Manian et al. 2006). Ratings were averaged into ethnic–ideal alignment scores: high scores reflect the belief that one's racial/ethnic identity overlaps with one's ideal future identity (0.66 ≤ α ≤ 0.91).

#### 2.2.2 School Belonging

On a scale from 1 (*strongly disagree*) to 7 (*strongly agree*), two items assessed school belonging (e.g., “I feel that I belong at school”; 0.52 ≤ α ≤ 0.68, 0.36 ≤ *r* ≤ 0.51).

#### 2.2.3 Markers of Academic Persistence

To report on their level of academic engagement, participants completed an adapted and reversed‐scored version of the Goal Disengagement Scale (Wrosch et al. 2003), so that high scores indicate high engagement instead of high goal disengagement (e.g., reversed: it's easy for me to reduce my effort toward my academic goals). Participants answered indicated their agreement to four items on a Likert scale from 1 (*strongly disagree*) to 4 (*strongly agree*). The present data displayed low reliability for some youths, which warrants caution (0.38 ≤ α ≤ 0.81, see also Table 1; we also examined possibilities to improve the measurement model, see supplements for related analyses presenting the second‐best model). Moreover, participants indicated whether they interpret difficulties as a signal of importance (often referred to, for short, as “difficulty‐as‐importance,” e.g., when I am working on a school task that feels difficult it means that the task is important; Fisher & Oyserman 2017) by reacting to four items on a Likert scale from 1 (*strongly disagree*) to 7 (*strongly agree*). In the present sample, this scale displayed good reliability (0.78 ≤ α ≤ 0.89).

**Table 1:** Demographic, descriptive, and reliability statistics, by race and gender.

	Latina girls	Latino boys	Black girls	Black boys
Demographics				
Age—M (SD)	14.00 (1.22)	13.99 (1.00)	14.37 (1.22)	13.90 (0.61)
*n*	94	84	38	30
Free lunch	58.5%	57.1%	60.5%	66.7%
No free lunch	27.7%	31.0%	2.6%	20.0%
Descriptive statistics				
Ethnic–ideal alignment—M (SD)	3.57 (0.74)	3.49 (0.85)	3.46 (1.07)	3.78 (1.09)
School belonging—M (SD)	5.20 (1.16)	5.22 (1.17)	4.97 (1.52)	5.42 (1.21)
Academic engagement—M (SD)	3.25 (0.53)	3.15 (0.52)	3.13 (0.53)	3.33 (0.74)
Difficulty‐as‐importance—M (SD)	5.14 (1.08)	4.92 (1.18)	4.65 (1.28)	4.78 (1.34)
Reliability statistics				
Ethnic–ideal alignment—*Cronbach α*	0.66	0.81	0.86	0.91
School belonging—*Cronbach α*	0.60	0.52	0.66	0.68
Academic engagement—*Cronbach α*	0.58	0.54	0.38	0.81
Difficulty‐as‐importance—*Cronbach α*	0.85	0.84	0.78	0.89
School belonging—*Pearson r*	0.46	0.36	0.50	0.51

*Note:* This table presents demographics, descriptive, and reliability statistics by race and gender. The participants who identified as Afro‐Latina, Afro‐Latino, or did not provide gender information are not included in these analyses.

## 3 Results

### 3.1 Analytical Plan

Analyses were conducted to examine, first, the possible direct and indirect roles of community resourcefulness and voice reflection conditions, through ethnic–ideal alignment (H1), tested separately for each marker of academic persistence, using PROCESS “Model 8” (Hayes 2022). We tested whether experimental conditions (dummy‐coded to contrast the voice reflection and the community resourcefulness reflection condition with the no‐reflection condition) yield significant direct and indirect effects on the two markers of academic persistence (academic engagement and difficulty‐as‐importance), through ethnic–ideal alignment. Furthermore, we tested whether gender moderates the conditions → ethnic–ideal alignment link, as well as the conditions → academic persistence link; and we tested for a statistical moderated mediation through bootstrap estimates.

Second, analyses were conducted to examine the possible direct and indirect roles of community resourcefulness and voice reflection conditions, through school belonging (H2), also tested separately for each marker of academic persistence, using PROCESS “Model 5” (Hayes 2022). We tested whether experimental conditions yield significant direct and indirect effects on the two markers of academic persistence, through school belonging; we further tested whether gender moderates the conditions → academic persistence link, and tested for statistical mediation through bootstrap estimates. Although we initially tested for a moderated mediation, we did not find evidence for one (see Model 8 results showing this pattern in Supporting Information S1); thus, we report only Model 5 results factoring in the role of gender on academic persistence in‐text.

### 3.2 Do Reflections on Racial/Ethnic Strengths Benefit Academic Persistence Through Ethnic–Ideal Alignment?

According to mediated moderation analyses, the community resourcefulness reflection condition increased academic engagement (see Table 2) and difficulty‐as‐importance (see Table 3) through its effect on ethnic–ideal alignment, and these effects operated distinctly for girls and boys. Indeed, adolescents in the community resourcefulness reflection condition (vs. neutral condition) reported higher ethnic–ideal alignment, a relationship moderated by gender. Simple effects reveal that the community resourcefulness reflection more effectively increased ethnic–ideal alignment for girls (*b* = 0.44, *p* = 0.014) than for boys (*b* = −0.15, *p* = 0.470). In turn, adolescent boys and girls who reported higher ethnic–ideal alignment tended to report higher academic engagement and to interpret difficulties as indicating importance.

**Table 2:** Model 8 linking reflections, ethnic–ideal alignment, and academic engagement.

	*b*	SE	*t*	*p*	95% CI
Regression predicting ethnic–ideal alignment: *F*(5, 242) = 1.874, *p* = 0.100, MSE = 0.770, *R* ^2^ = 0.037					
Intercept	3.29	0.13	25.14	< 0.001	[3.03, 3.55]
Voice (a‐path)	0.26	0.20	1.34	0.182	[−0.13, 0.65]
Community resourcefulness (a‐path)	**0.44**	**0.18**	**2.48**	**0.014**	**[0.09, 0.80]**
Gender	**0.46**	**0.19**	**2.39**	**0.018**	**[0.08, 0.84]**
Voice × Gender	**−0.60**	**0.28**	**−2.17**	**0.031**	**[−1.15, −0.06]**
Community resourcefulness × Gender	**−0.59**	**0.27**	**−2.17**	**0.031**	**[−1.13, −0.05]**
Regression predicting academic engagement: *F*(6, 241) = 2.420, *p* = 0.027, MSE = 0.301, *R* ^2^ = 0.057					
Intercept	2.84	0.16	18.28	< 0.001	[2.53, 3.14]
Voice (c'‐path)	−0.05	0.12	−0.41	0.686	[−0.30, 0.19]
Community resourcefulness (c’‐path)	0.12	0.11	1.03	0.306	[−0.11, 0.34]
Ethnic–ideal alignment (b‐path)	**0.10**	**0.04**	**2.49**	**0.014**	**[0.02, 0.18]**
Gender	−0.18	0.12	−1.47	0.142	[−0.42, 0.06]
Voice × Gender	**0.35**	**0.18**	**2.01**	**0.045**	**[0.01, 0.70]**
Community resourcefulness × Gender	0.07	0.17	0.42	0.677	[−0.27, 0.41]

*Note:* Bold values are statistically significant results with *p* < 0.05. Bootstrap analyses with 5000 samples revealed a mediation effect moderated by gender of the community resourcefulness reflection through ethnic–ideal alignment on academic engagement (*b* = −0.059, SE = 0.040, CI_95%_ [−0.150, −0.001]), but none through the voice reflection (*b* = −0.060, SE = 0.043, CI_95%_ [0.164, −0.000]).

**Table 3:** Model 8 linking reflections, ethnic–ideal alignment, and difficulty‐as‐importance.

	*b*	SE	*t*	*p*	95% CI
Regression predicting ethnic–ideal alignment: *F*(5, 237) = 1.764, *p* = 0.121, MSE = 0.759, *R* ^2^ = 0.036					
Intercept	3.34	0.13	25.12	< 0.001	[3.08, 3.60]
Voice (a‐path)	0.21	0.20	1.01	0.282	[−0.18, 0.61]
Community resourcefulness (a‐path)	**0.43**	**0.18**	**2.37**	**0.019**	**[0.07, 0.79]**
Gender	**0.41**	**0.19**	**2.12**	**0.035**	**[0.03, 0.79]**
Voice × Gender	**−0.55**	**0.28**	**−1.99**	**0.048**	**[−1.10, −0.01]**
Community resourcefulness × Gender	**−0.55**	**0.27**	**−2.00**	**0.047**	**[−1.09, −0.01]**
Regression predicting difficulty‐as‐importance: *F*(6, 236) = 2.577, *p* = 0.019, MSE = 1.362, *R* ^2^ = 0.061					
Intercept	3.76	0.34	11.04	< 0.001	[3.09, 4.43]
Voice (c’‐path)	0.29	0.27	1.08	0.281	[−0.24, 0.81]
Community resourcefulness (c’‐path)	0.34	0.25	1.39	0.165	[−0.14, 0.83]
Ethnic–ideal alignment (b‐path)	**0.29**	**0.09**	**3.30**	**< 0.001**	**[0.12, 0.46]**
Gender	0.11	0.26	0.41	0.682	[−0.41, 0.62]
Voice × Gender	−0.18	0.37	−0.48	0.635	[−0.91, 0.56]
Community resourcefulness × Gender	−0.45	0.37	−1.22	0.225	[−1.19, 0.28]

*Note:* Bold values are statistically significant results with *p* < 0.05. Bootstrap analyses with 5000 samples revealed a mediation effect moderated by gender of the community resourcefulness reflection through ethnic–ideal alignment on difficulty‐as‐importance (*b* = −0.157, SE = 0.094, CI_95%_ [−0.362, −0.002]), but none through the voice reflection (*b* = −0.158, SE = 0.105, CI_95%_ [0.398, 0.003]).

Bootstrap analyses with 5000 samples revealed mediation effects moderated by gender of the community resourcefulness reflection on academic engagement (*b*_*ab‐*path_ = −0.059, SE = 0.040, CI_95%_ [−0.150, −0.001]) and on difficulty‐as‐importance (*b*_*ab‐*path_ = −0.157, SE = 0.094, CI_95%_ [−0.362, −0.002]). Specifically, Black and Latina girls assigned to the community resourcefulness reflection condition (vs. the no‐reflection condition) reported higher ethnic–ideal alignment, which was in turn associated with higher academic engagement (*b*_*ab‐*path_ = 0.044, SE = 0.027, CI_95%_ [.002, 0.106]); for Black and Latino boys, no mediation was found (*b*_*ab‐*path_ = −0.015, SE = 0.023, CI_95%_ [−0.069, 0.024]). Similarly, Black and Latina girls assigned to the community resourcefulness reflection condition (vs. the no‐reflection condition) reported higher ethnic–ideal alignment, which was in turn associated with higher interpretation of difficulty as importance (*b*_*ab‐*path_ = 0.123, SE = 0.064, CI_95%_ [0.020, 0.268]); no mediation was found for Black and Latino boys (*b*_*ab‐*path_ = −0.034, SE = 0.061, CI_95%_ [−0.166, 0.082]).

**Figure 1:**
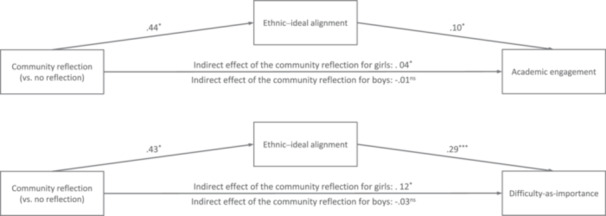
Main findings from the two models testing H1: The community resourcefulness reflection (vs. no reflection) indirectly predicted markers of academic persistence, through effects on ethnic?ideal alignment for Black and Latina girls, not for boys; there was no indirect effect of the voice reflection.

In contrast, adolescents in the voice reflection condition did not differ in terms of ethnic–ideal alignment, on average, from adolescents in the no‐reflection condition. Bootstrap analyses did not reveal mediation effects of school belonging through the voice reflection condition, whether on academic engagement or difficulty‐as‐importance for boys or for girls, and no moderated mediation either. Thus, the community resourcefulness reflection condition indirectly increased markers of academic persistence through ethnic–ideal alignment, an effect driven by Black and Latina girls (Figure 1).

### 3.3 Do Reflections on Racial/Ethnic Strengths Benefit Academic Persistence Through School Belonging?

According to mediation analyses, the voice reflection condition increased both academic engagement (see Table 4) and interpretation of difficulty as important (see Table 5), through its effect on school belonging. Specifically, the voice reflection condition (vs. no‐reflection condition) increased school belonging, whereas the community resourcefulness reflection condition did not. In turn, higher school belonging was associated with higher academic engagement and a higher tendency to interpret difficulties as indicating importance.

**Table 4:** Model 5 linking reflections, ethnic–ideal alignment, and academic engagement.

	*b*	SE	*t*	*p*	95% CI
Regression predicting school belonging: *F*(2, 248) = 5.990, *p* = 0.003, MSE = 1.449, *R* ^2^ = 0.046					
Intercept	4.89	0.13	37.25	< 0.001	[4.63, 5.15]
Voice (a‐path)	**0.65**	**0.19**	**3.46**	**< 0.001**	**[0.28, 1.02]**
Community resourcefulness (a‐path)	0.31	0.18	1.71	0.089	[−0.05, 0.68]
Regression predicting academic engagement: *F*(6, 244) = 7.429, *p* < 0.001, MSE = 0.270, *R* ^2^ = 0.154					
Intercept	2.36	0.15	15.30	< 0.001	[2.05, 2.66]
Voice (c’‐path)	−0.15	0.12	−1.23	0.219	[−0.38, 0.09]
Community resourcefulness (c’‐path)	0.12	0.11	1.10	0.271	[−0.09, 0.32]
School belonging (b‐path)	**0.17**	**0.03**	**6.02**	**< 0.001**	**[0.11, 0.22]**
Gender	−0.15	0.11	−1.27	0.204	[−0.37, 0.08]
Voice × Gender	**0.32**	**0.16**	**1.97**	**0.050**	**[0.00, 0.64]**
Community resourcefulness × Gender	0.03	0.16	−0.17	0.866	[−0.35, 0.29]

*Note:* Bold values are statistically significant results with *p* < 0.05. According to bootstrap analyses with 5000 samples, school belonging mediated the indirect effect of the voice reflection condition on academic engagement (*b*_*ab*‐path_ = 0.11, SE = 0.03, CI_95%_ [0.04, 0.18]), but not the effect of the community resourcefulness reflection condition (*b*_*ab*‐path_ = 0.05, SE = 0.03, CI_95%_ [−0.01, 0.12]).

**Table 5:** Model 5 linking reflections, ethnic–ideal alignment, and difficulty‐as‐importance.

	*b*	SE	*t*	*p*	95% CI
Regression predicting school belonging: *F*(2, 242) = 5.313, *p* = 0.006, MSE = 1.456, *R* ^2^ = 0.042					
Intercept	4.92	0.13	36.93	< 0.001	[4.66, 5.18]
Voice (a‐path)	**0.62**	**0.19**	**3.25**	**< 0.001**	**[0.24, 0.99]**
Community resourcefulness (a‐path)	0.25	0.19	1.35	0.180	[−0.12, 0.62]
Regression predicting difficulty‐as‐importance: *F*(6, 238) = 8.254, *p* < 0.001, MSE = 1.194, *R* ^2^ = 0.172					
Intercept	2.82	0.33	8.53	< 0.001	[2.17, 3.47]
Voice (c’‐path)	0.04	0.25	0.14	0.886	[−0.46, 0.53]
Community resourcefulness (c’‐path)	0.38	0.23	1.71	0.089	[−0.06, 0.83]
School belonging (b‐path)	**0.39**	**0.06**	**6.70**	**< 0.001**	**[0.28, 0.51]**
Gender	0.18	0.24	0.72	0.471	[−0.30, 0.65]
Voice × Gender	−0.22	0.35	−0.64	0.525	[−0.90, 0.46]
Community resourcefulness × Gender	**−0.72**	**0.35**	**−2.10**	**0.037**	**[−1.40, −0.05]**

*Note:* Bold values are statistically significant results with *p* < 0.05. According bootstrap analyses with 5000 samples, school belonging mediated the indirect effect of the voice reflection condition on difficulty‐as‐importance (*b*_*ab‐*path_ = 0.24, SE = 0.09, CI_95%_ [0.08, 0.44]), but not the effect of the community resourcefulness reflection condition (*b*_*ab‐*path_ = 0.10, SE = 0.08, CI_95%_ [−0.05, 0.26]).

**Figure 2:**
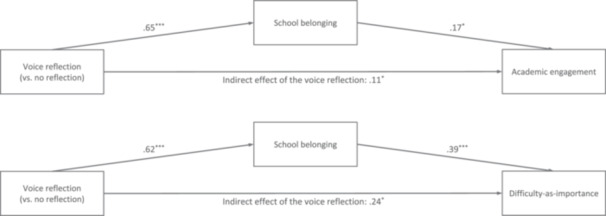
Main findings from the two models testing H2: The voice reflection (vs. no reflection) indirectly predicted markers of academic persistence, through its effect on school belonging; there was no indirect effect of the community resourcefulness reflection.

According to bootstrap analyses with 5000 samples, school belonging mediated the indirect effect of the voice reflection condition on academic engagement (*b*_*ab‐*path_ = 0.11, SE = 0.03, CI_95%_ [0.04, 0.18]), but not the effect of the community resourcefulness reflection condition (*b*_*ab‐*path_ = 0.05, SE = 0.03, CI_95%_ [−0.01, 0.12]). Mirroring those findings, additional bootstrap analyses indicated that school belonging mediated the indirect effect of the voice reflection condition on difficulty‐as‐importance (*b*_*ab‐*path_ = 0.24, SE = 0.09, CI_95%_ [0.08, 0.44]), but not the effect of the community resourcefulness reflection condition (*b*_*ab‐*path_ = 0.10, SE = 0.08, CI_95%_ [−0.05, 0.26]). Thus, the voice reflection (but not the community resourcefulness reflection) indirectly increased academic persistence for Black and Latine adolescents insofar as it increased school belonging (see Figure 2).

## 4 Discussion

During adolescence, Black and Latine youth explore their racial/ethnic identity and future aspirations, including career possibilities. Prior research and theory indicate that using race‐conscious strength‐based approaches within academic contexts could change how adolescents view their racial/ethnic experiences in relation to school and aspirations, with implications for their academic and career trajectories. Using a randomized controlled design, the current study investigated whether two types of strength‐based reflections would increase Black and Latine adolescents' ethnic–ideal alignment and school belonging and, in turn, their academic persistence (assessed with two markers: academic engagement and difficulty‐as‐importance). Adolescents who completed the community resourcefulness reflection were presented with strengths and advice from Black and Latine people and considered their own aspirations and obstacles in pursuing them. Adolescents who completed the voice reflection considered how their own strengths and perspectives could play a positive role if placed in a context where their group was underrepresented.

The present findings show that community resourcefulness and voice reflections can indirectly benefit academic persistence, that they operate through different mechanisms, and that their effects can vary depending on gender. Black and Latina girls (but not boys) who completed the community resourcefulness reflection experienced greater ethnic–ideal alignment (but not school belonging). In turn, Black and Latina girls' ethnic–ideal alignment was associated with greater academic engagement and interpretation of school difficulty as indicating school importance. Conversely, both Black and Latine boys and girls who completed the voice reflection experienced a greater sense of school belonging (but not ethnic–ideal alignment), which, in turn, was associated with greater academic engagement and difficulty‐as‐importance. Thus, the community resourcefulness reflection may be especially effective among Black and Latina girls, insofar as the reflection increases their ethnic–ideal alignment. Relatedly, the voice reflection has the potential to benefit academic persistence indirectly insofar as reflections elicit school belonging for Black and Latine boys and girls.

These findings have implications for adolescents of color's developmental trajectories. There is increased empirical and theoretical focus on the ways in which racial and ethnic experiences can shape the trajectory of adolescents of color, and on the ways they can benefit them (Hoffman and Umaña‐Taylor 2023; Jones and Neblett 2016; Rogers et al. 2021). In this context, the present study provides an experimental test for how two types of reflections and the messages they convey can affect academic persistence, school belonging, as well as adolescents' identities. Extrapolating from these findings, it is possible to imagine that repeated messages about racial/ethnic identities aligning with their ideal future identities through adolescents' development could nurture pathways marked by increased academic persistence. Relatedly, it is possible that specific turning points during adolescence are particularly central in identity development and, thus, such messages may be particularly meaningful during specific periods.

One remaining question for future research is understanding whether and how strength‐based approaches like community resourcefulness reflection can elicit school belonging among Black and Latine adolescents. The community resourcefulness reflection counters negative and narrow narratives about Black and Latine communities by inviting reflections on positive actions taken by other Black and Latine people to overcome obstacles, but it does not explicitly guide students to draw connections between their identity/background and their school community. Indeed, when adolescents of color reflect meaningfully on the important people in their lives (e.g., when engaging in culturally relevant affirmations that focus on family), they perform better academically (Covarrubias et al. 2016), but they do not necessarily experience a greater connection with academic contexts. In this way, the current research contributes to the literature on strength‐based approaches by providing further credence to the idea that connecting their racial/ethnic background and school is beneficial for adolescents of color.

Intersectional considerations guided us in examining how strength‐based reflections differentially affect Black and Latine boys and girls during adolescence—a strength in this study. One possibility is that adolescent girls of color may experience more systemically imposed challenges in forming strong connections between their racial/ethnic identity and their ideal future identity; as a result, they may benefit the most from strength‐based tasks like the community resourcefulness reflection implemented in this study. Another possibility is that the community resourcefulness reflection evoked Black and Latina girls' communal orientation, making it particularly beneficial because it tapped into a central value to many. Girls and women of color have historically had to contend with the systemically induced tension associated with holding important familial responsibilities while pursuing their personal goals (e.g., Ovink 2014). For these reasons, Black and Latina adolescent girls may find it harder to connect their race/ethnicity and their ideal future identity; therefore, they may benefit the most from strength‐based reflection activities.

Yet, the current design did not allow testing a determinant mechanism or examining formally why the community resourcefulness reflection worked for Black and Latine adolescent girls but not boys, in the present context. This represents an avenue for future research that will provide insight into strength‐based approaches that benefit both Black and Latine boys and girls during adolescence. In terms of limitations, the study also was underpowered to examine moderated mediations, and its design allowed for examining the causal influence of the reflections, but not of mediators. This is, in part, because the subsamples of Black boys and Black girls were both small. Small samples are more likely to create Type I errors and less likely to capture the experiences of whole populations accurately, such as the populations of Black boys and girls in the U.S. While conducting randomized controlled experiments with large numbers of young people of color is challenging, researchers should exert caution when considering whether findings identified with small samples generalize. Therefore, the current design represents a meaningful first step in examining the role of racial/ethnic strength‐based reflections, but cannot rule out several alternative explanations.

Other limitations of the present study include the measurement model. In the present case, the school belonging scale included only two items, which is common when measuring school belonging, but nonetheless a limitation. Also, the academic engagement scale was not reliable, which suggests cautious interpretation since some students could have misunderstood the items; yet this indicator yielded findings consistent with the other marker, difficulty‐as‐importance, that showed acceptable reliability. Future studies aiming to replicate and extend these results could use more items to assess school belonging and academic engagement, to improve reliability. Moreover, adolescents were the sole respondents in the present study; opportunities to triangulate the results with reports from their teachers, with perspectives from their families, or with official school records would strengthen the findings of future studies examining similar tasks. Therefore, while the present research shows meaningfully the potential benefits of racial/ethnic strength‐based tasks for Black and Latine adolescents, future research can continue testing their potential and their effects, including by varying measures and triangulating sources.

This study also has implications for carefully designed interventions that target specific challenges that adolescents of color may be facing on their educational trajectories. On the one hand, adolescents who are uncertain that they belong in academic settings may benefit from reflecting on their own strengths and perspectives when considering how they could play a positive role because of their unique point of view if they were underrepresented in a particular context (e.g., the voice reflection). On the other hand, adolescent girls who find it challenging to make connections between their racial/ethnic identity and their ideal future identity may benefit from reflecting on the strategies implemented by other people of color to overcome academic setbacks. Strength‐based programs bringing several activities together could provide the opportunity for adolescents to reflect on the strategies used by their communities while also being reminded of their unique assets and the important role they can play in their milieux. Future research can investigate whether combining such activities can contribute to adolescents' health and well‐being in addition to their motivation. It is important that academic achievement and motivation not come at a cost to the health and well‐being of young adolescents pursuing their goals (Destin et al. 2024). One way to facilitate Black and Latine adolescents' academic success and well‐being is by providing them the opportunity to draw from and leverage the cultural capital associated with their identity. Black and Latine adolescents have unique knowledge and strengths that they have developed because of their lived experiences; they can also draw from the knowledge and strengths of their communities—one of their greatest assets.

## Author Contributions

Both authors contributed to the research project's conceptualization and to method designs, as well as to writing, reviewing, and editing the manuscript, with R.D. leading the project and contributing more. R.D. also acquired funds and provided resources to support the project, conducted formal analyses, and created visualization tools to represent the data. Both R.D. and Character Lab contributed to project administration, data curation, and data collection.

## Ethics Statement

Data were collected with Character Lab Research Network (CLRN), a consortium of scientists and school partners that facilitates research in U.S. schools. After obtaining ethics approval for the study, CLRN approved the project and its protocol, then matched the project with school partners and determined the sample size based on the availability of the student population among partners and concomitant projects. In each partnered school, students access studies through Qualtrics links set on computers. CLRN then deidentified the data before making it accessible. Ethics approval was also provided by Northwestern University, then the affiliation of the first author (STU00211126). Students who participated in the study, and their parents, provided consent.

## Conflicts of Interest

The authors declare that there are no potential conflicts of interest with respect to the research, authorship, and/or publication of this article.

## Supporting information


**Table I:** Statistics for 2‐item academic engagement scale, by race and gender. **Table II:** Model 8 linking reflections, ethnic–ideal alignment, and 2‐item academic engagement. **Table III:** Model 5 linking reflections, ethnic–ideal alignment, and 2‐item academic engagement. **Table IV:** Model 8 testing mediation of reflections, belonging, and academic engagement moderated by gender. **Table V:** Model 8 testing mediation of reflections, belonging, and interpretation of difficulty as signaling importance moderated by gender.

## Data Availability

Data and code are available upon request. Materials are available as Supporting Information S1.
