# Supplementary material for: Community and Voice: Emphasizing Black and Latine Adolescents' Strengths Promotes Identity Alignment, Belonging, and Academic Persistence
Source: J Adolesc. 2025 Sep 9;98(1):175–86. doi: 10.1002/jad.70049 (PMC12780650; doi:10.1002/jad.70049)
Supplement: Supplementary file 1 — Table I: Statistics for 2‐item academic engagement scale, by race and gender. Table II: Model 8 linking reflections, ethnic–ideal alignment, and 2‐item academic engagement. Table III: Model 5 linking reflections, ethnic–ideal alignment, and 2‐item academic engagement. Table IV: Model 8 testing mediation of reflections, belonging, and academic engagement moderated by gender. Table V: Model 8 testing mediation of reflections, belonging, and interpretation of difficulty as signaling importance moderated by gender. [file JAD-98-175-s001.docx]

**Supplemental results – Alternate measurement model**

In an attempt to improve the measurement model for the academic engagement scale because the original four-item scale displayed lower reliability than expected, analyses were conducted with the two strongest items. Here are the

**Table I: Statistics for 2-item academic engagement scale, by race and gender**

|  | *Latina girls* | *Latino boys* | *Black girls* | *Black boys* | ***Total*** |
| --- | --- | --- | --- | --- | --- |
| 2-item academic engagement -- *M (SD)* | 2.40 (.72) | 2.30 (.74) | 2.29 (.63) | 2.43 (.85) | **2.34 (.72)** |
| 2-item academic engagement -- *Cronbach α* | .73 | .61 | .22 | .78 | **.55** |
| 2-item academic engagement -- *Pearson r* | .60^***^ | .45^***^ | .13 | .65^***^ | **.38^***^** |
|  |  |  |  |  |  |

**Table II: Model 8 linking reflections, ethnic–ideal alignment, and 2-item** **academic engagement**

|  | *b* | *SE* | *t* | | *p* | *95% C.I.* | |  |
| --- | --- | --- | --- | --- | --- | --- | --- | --- |
| ***Regression predicting ethnic–ideal alignment: F(5, 242) = 1.874, p=.100, MSE=.770, R^2^=.037*** | | | | | | | | |
| Intercept | 3.29 | .13 | 25.14 | <.001 | | [3.03, 3.55] |  |  |
| Voice (*a-path)* | .27 | .20 | 1.34 | .182 | | [-.13, .65] |  |  |
| **Community resourcefulness^*^ (*a-path)*** | **.44** | **.18** | **2.48** | **.014** | | **[.09, .80]** |  |  |
| **Gender** | **.46** | **.19** | **2.39** | **.018** | | **[.08, .84]** |  |  |
| **Voice × Gender** | **-.60** | **.28** | **-2.17** | **.031** | | **[-1.15, -.06]** |  |  |
| **Community resourcefulness × Gender** | **-.59** | **.27** | **-2.17** | **.031** | | **[-1.13, -.05]** |  |  |
| ***Regression predicting 2-item academic engagement: F(6, 241) = 1.752, p=.110, MSE=.518, R^2^=.042*** | | | | | | | | |
| Intercept | 1.91 | .20 | 9.35 | | <.001 | [1.50, 2.31] | |  |
| Voice (*c’-path)* | .02 | .16 | .10 | | .923 | [-.31, .34] | |  |
| Community resourcefulness (*c’-path)* | .05 | .15 | .30 | | .763 | [-.25, .34] | |  |
| **Ethnic–ideal alignment (*b-path)*** | **.13** | **.05** | **2.40** | | **.017** | **[.02, .23]** | |  |
| Gender | -.22 | .16 | -1.39 | | .166 | [-.54, .09] | |  |
| Voice × Gender | .35 | .23 | 1.51 | | .133 | [-.11, .80] | |  |
| Community resourcefulness × Gender | .14 | .23 | .62 | | .538 | [-.31, .59] | |  |
|  |  |  |  | |  |  | |  |
|  | | | | | | | |  |

**Table III. Model 5 linking reflections, ethnic–ideal alignment, and 2-item academic engagement**

|  | *b* | *SE* | *t* | | *p* | *95% C.I.* | |  |
| --- | --- | --- | --- | --- | --- | --- | --- | --- |
| ***Regression predicting belonging: F(2, 248) = 5.990, p=.003, MSE=1.449, R^2^=.046*** | | | | | | | | |
| Intercept | 4.89 | .13 | 37.25 | <.001 | | [4.63, 5.15] |  |  |
| **Voice^***^ (*a-path)*** | **.65** | **.19** | **3.46** | **<.001** | | **[.28, 1.02]** |  |  |
| Community resourcefulness (*a-path)* | .31 | .18 | 1.71 | .089 | | [-.05, .68] |  |  |
| ***Regression predicting 2-item academic engagement: F(6, 244) = 6.946, p<.001, MSE=.460, R^2^=.146*** | | | | | | | | |
| Intercept | 1.26 | .20 | 6.27 | | <.001 | [.86, 1.66] | |  |
| Voice (*c’-path)* | -.12 | .15 | -.75 | | .452 | [-.42, .19] | |  |
| Community resourcefulness (*c’-path)* | .05 | .14 | .34 | | .743 | [-.22, .32] | |  |
| **Belonging ^***^ (*b-path)*** | **.22** | **.04** | **6.06** | | **<.001** | **[.15, .29]** | |  |
| Gender | -.18 | .15 | -1.24 | | .216 | [-.48, .11] | |  |
| Voice × Gender | .31 | .21 | 1.46 | | .144 | [-.11, 73] | |  |
| Community resourcefulness × Gender | .01 | .21 | .04 | | .971 | [-.41, .42] | |  |
|  |  |  |  | |  |  | |  |
|  | | | | | | | |  |

**Supplemental results – Moderated Mediation with Gender and Belonging**

**Table IV: Model 8 testing mediation of reflections, belonging, and academic engagement moderated by gender**

|  | *b* | *SE* | *t* | | *p* | *95% C.I.* | |  |
| --- | --- | --- | --- | --- | --- | --- | --- | --- |
| ***Regression predicting belonging: F(5, 245) = 3.040, p=.011, MSE=1.448, R^2^=.058*** | | | | | | | | |
| Intercept | 4.84 | .18 | 27.26 | <.001 | | [4.49, 5.19] |  |  |
| **Voice^**^ (*a-path)*** | **.79** | **.27** | **2.93** | **.004** | | **[.26, 1.32]** |  |  |
| Community resourcefulness (*a-path)* | .20 | .24 | .83 | .409 | | [-.28, .68] |  |  |
| Gender | .12 | .26 | .47 | .640 | | [-.40, .64] |  |  |
| Voice × Gender | -.27 | .38 | -.73 | .469 | | [-1.02, .47] |  |  |
| Community resourcefulness × Gender | .31 | .37 | .83 | .408 | | [-.43, 1.04] |  |  |
| ***Regression predicting academic engagement: F(6, 244) = 7.429, p<.001, MSE=. 270, R^2^=. 154*** | | | | | | | | |
| Intercept | 2.35 | .15 | 15.30 | | <.001 | [2.05, 2.66] | |  |
| Voice (*c’-path)* | -.15 | .12 | -1.23 | | .219 | [-.38, .09] | |  |
| Community resourcefulness (*c’-path)* | .12 | .10 | 1.10 | | .271 | [-.09, .32] | |  |
| **Belonging ^***^ (*b-path)*** | **.17** | **.03** | **6.02** | | **<.001** | **[.11, .22]** | |  |
| Gender | -.15 | .11 | -1.27 | | .204 | [-.37, .08] | |  |
| Voice × Gender | .32 | .16 | 1.97 | | .050 | [.00, .64] | |  |
| Community resourcefulness × Gender | -.03 | .16 | -.17 | | .866 | [-.35, .29] | |  |
|  |  |  |  | |  |  | |  |
| Indices of moderated mediation indicate that gender do not moderate the possible indirect effects through belonging of the voice condition (*b* = -.046, SE = .064, CI_95%_ [-.175, .083]) nor of the community resourcefulness condition (*b* = .051, SE = .064, CI_95%_ [-.073, .180]). | | | | | | | |  |

**Table V: Model 8 testing mediation of reflections, belonging, and interpretation of difficulty as signaling importance moderated by gender**

|  | *b* | *SE* | *t* | | *p* | *95% C.I.* | |  |
| --- | --- | --- | --- | --- | --- | --- | --- | --- |
| ***Regression predicting belonging: F(5, 245) = 3.040, p=.011, MSE=1.448, R^2^=.058*** | | | | | | | | |
| Intercept | 4.89 | .18 | 26.88 | <.001 | | [4.53, 5.24] |  |  |
| **Voice^**^ (*a-path)*** | **.74** | **.27** | **2.72** | **.007** | | **[.20, 1.28]** |  |  |
| Community resourcefulness (*a-path)* | .10 | .25 | .42 | .676 | | [-.39, .59] |  |  |
| Gender | .07 | .27 | .28 | .781 | | [-.45, .60] |  |  |
| Voice × Gender | -.24 | .38 | -.62 | .536 | | [-.99, .52] |  |  |
| Community resourcefulness × Gender | .39 | .38 | 1.03 | .305 | | [-.36, 1.14] |  |  |
| ***Regression predicting interpretation of difficulty: F(6, 238) = 8.254, p<.001, MSE=1.194, R^2^=. 172*** | | | | | | | | |
| Intercept | 2.82 | .33 | 8.53 | | <.001 | [2.17, 3.47] | |  |
| Voice (*c’-path)* | .04 | .25 | .14 | | .886 | [-.46, .53] | |  |
| Community resourcefulness (*c’-path)* | .38 | .23 | 1.71 | | .089 | [-.06, .83] | |  |
| **Belonging ^***^ (*b-path)*** | **.39** | **.06** | **6.70** | | **<.001** | **[.28, .51]** | |  |
| Gender | .18 | .24 | .72 | | .471 | [-.30, .65] | |  |
| Voice × Gender | -.22 | .35 | -.64 | | .525 | [-.90, .46] | |  |
| **Community resourcefulness × Gender** | **-.72** | **.35** | **-2.10** | | **.037** | **[-1.40, -.05]** | |  |
|  |  |  |  | |  |  | |  |
| Indices of moderated mediation indicate that gender do not moderate the possible indirect effects through belonging of the voice condition (*b* = -.093, SE = .151, CI_95%_ [-.399, .193]) nor of the community resourcefulness condition (*b* = .153, SE = .151, CI_95%_ [-.140, .459]). | | | | | | | |  |
